# The Enhancement of Mg Corrosion Resistance by Alloying Mn and Laser-Melting

**DOI:** 10.3390/ma9040216

**Published:** 2016-03-23

**Authors:** Youwen Yang, Ping Wu, Qiyuan Wang, Hong Wu, Yong Liu, Youwen Deng, Yuanzhuo Zhou, Cijun Shuai

**Affiliations:** 1 State Key Laboratory of High Performance Complex Manufacturing, Central South University, Changsha 410083, China; yangyouwen@csu.edu.cn (Y.Y.); zyz420357155@csu.edu.cn (Y.Z.); 2 College of Chemistry, Xiangtan University, Xiangtan 411105, China; pingwu@xtu.edu.cn; 3 Department of Emergency Medicine, 2nd Xiangya Hospital of Central South University, Changsha 410083, China; idletwo@gmail.com (Q.W.); dengjunjiejj@csu.edu.cn (Y.D.); 4 State Key Laboratory for Powder Metallurgy, Central South University, Changsha 410083, China; hwucsu@csu.edu.cn (H.W.); yonliu@csu.edu.cn (Y.L.)

**Keywords:** corrosion resistance, Mg-Mn alloy, laser-melting, microstructure, mechanical properties

## Abstract

Mg has been considered a promising biomaterial for bone implants. However, the poor corrosion resistance has become its main undesirable property. In this study, both alloying Mn and laser-melting were applied to enhance the Mg corrosion resistance. The corrosion resistance, mechanical properties, and microstructure of rapid laser-melted Mg-*x*Mn (*x* = 0–3 wt %) alloys were investigated. The alloys were composed of dendrite grains, and the grains size decreased with increasing Mn. Moreover, Mn could dissolve and induce the crystal lattice distortion of the Mg matrix during the solidification process. Mn ranging from 0–2 wt % dissolved completely due to rapid laser solidification. As Mn contents further increased up to 3 wt %, a small amount of Mn was left undissolved. The compressive strength of Mg-Mn alloys increased first (up to 2 wt %) and then decreased with increasing Mn, while the hardness increased continuously. The refinement of grains and the increase in corrosion potential both made contributions to the enhancement of Mg corrosion resistance.

## 1. Introduction

Recently, Mg has attracted widespread attention as new biodegradable material for bone implants due to its inherent degradability, proper mechanical properties, and good biocompatibility [1,2,3]. However, the corrosion rate of Mg in body fluid is too fast to allow the Mg-based implants to maintain its mechanical strength during the recovery of injured bone (at least three months) [4,5]. Moreover, the fast corrosion of Mg results in the release of a large amount of hydrogen gas in body fluid, which is detrimental for the host tissue [6].

Alloying is an effective method to enhance the Mg corrosion resistance. Biodegradable Mg-Ca alloys have been developed and have demonstrated an improved corrosion behavior both in simulated body fluid (SBF) and Hank’s solutions [7,8]. The Mg-Sr alloy system was designed, and the results indicated that the addition of 2 wt % Sr improved the Mg corrosion resistance [9]. Other Mg alloys, such as AZ31, AZ91, WE43, and LAE442, have also been reported to have improved Mg corrosion resistance [10,11].

Rapid solidification is also an effective method to enhance the Mg corrosion resistance. Laser-melting is a typical rapid solidification technology involving a cooling rate above 10^5^ K/s [12,13]. The high cooling rate will prevent the growth of grains for finer crystalline structure, which has a beneficial effect on enhancing the Mg corrosion resistance [14,15]. Meanwhile, rapid laser-melting is able to homogenize the compositional distribution, thus limiting the local cell action caused by the accumulation of cathodic phases [16]. Furthermore, rapid laser-melting could increase the solid solubility of alloying elements, such as Mn, Al, and Cr, promoting the formation of more protective and self-healing films [16]. Mn is an essential element in human bone, and its average daily intake of an adult is about 5–10 mg [17]. Furthermore, the *in vivo* test revealed that it promoted cell proliferation and bone regeneration [18]. Furthermore, Gu *etc.* [19] have studied the mechanical properties, corrosion properties, and *in vitro* biocompatibilities of as-cast Mg-1Mn (wt %) alloys, which suggests that the addition of alloying element Mn could improve the strength and corrosion resistance of Mg.

In this paper, both alloying Mn and rapid laser-melting were applied to enhance the Mg corrosion resistance. Basing on the Mg–Mn binary phase diagram, the solubility of Mn in Mg at room temperature is below 1 wt %, and increases to ~2 wt % at 750 °C [20].Thus, we designed a series of Mg-Mn alloys containing Mn from 0–3 wt %. The corrosion resistance, mechanical properties, and microstructure of rapid laser-melted Mg-Mn alloys were investigated.

## 2. Results and Discussion

### 2.1. Microstructure Characteristics

**Figure 1:**
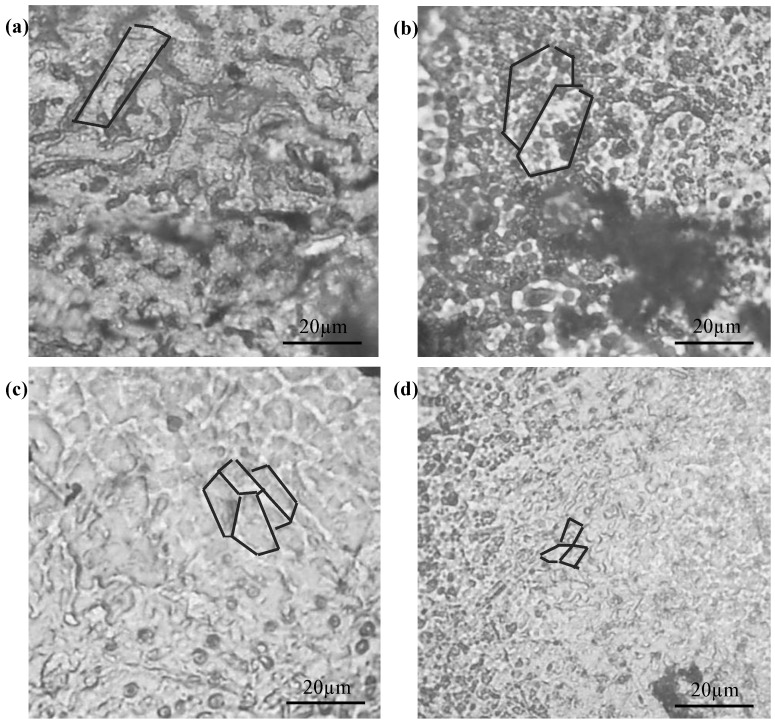
Crystalline structures of laser-melted Mg-*x*Mn alloys: (**a**) pure Mg; (**b**) Mg-1Mn alloy; (**c**) Mg-2Mn alloy; (**d**) Mg-3Mn alloy.

The Mg-*x*Mn alloys consisted of dendrite grains whose sizes decreased with increasing Mn (Figure 1). Pure Mg presented relatively large dendrite (~20 µm) (Figure 1a). As Mn increased to 2 wt %, the grains reduced to ~15 µm (Figure 1c). With Mn further increasing up to 3 wt %, the grains were further refined (~10 µm) (Figure 1d). Obviously, rapid laser-melted Mg alloys had finer grains compared to powder metallurgy processed Mg alloys with a typical grain size of 50 µm [21,22] and casted Mg alloys with larger grain sizes [7].

**Figure 2:**
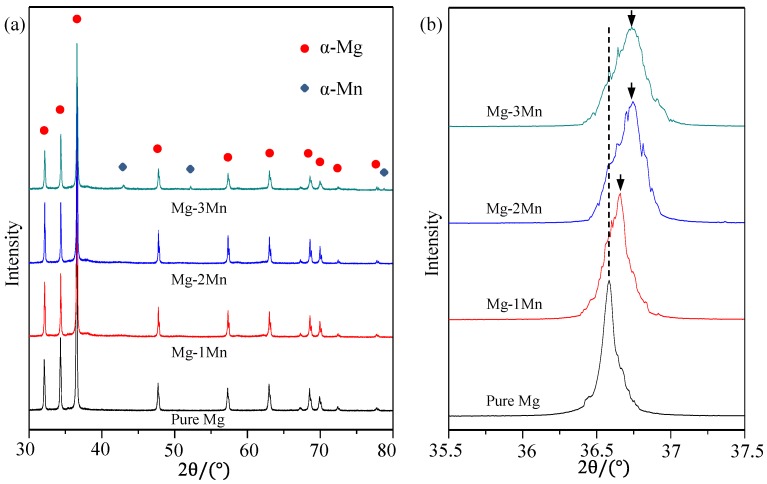
(**a**) X-ray diffractometer (XRD) spectrum of rapid laser-melted Mg-Mn alloy obtained over 30°–80°; (**b**) XRD spectra in the vicinity of the standard diffraction peak of Mg 2θ = 36.53°.

The phase composition of Mg-*x*Mn alloys was tested by X-ray diffractometer (XRD) within a wide 2θ range (30°–80°) (Figure 2a). The results showed that only diffraction peaks of the Mg phase were detected in the Mg-1Mn and Mg-2Mn alloys, while the diffraction peaks of both Mg and Mn phases were detected in the Mg-3Mn alloy. It indicated that Mn ranging from 0 to 2 wt % completely dissolved in the Mg matrix due to the rapid solidification and formed supersaturated solid solutions. However, 3 wt % Mn exceeded the solubility limit ~2 wt % at 750 °C (melten pool temperature); thus, a small amount of Mn phase was left undissolved in the Mg matrix after the rapid solidification.

The XRD characterization within a small 2θ range (35.5°–37.5°) (Figure 2b) revealed that the location of the diffraction peak for Mg apparently changed with increasing Mn. The standard diffraction peak of Mg (2θ = 36.53°) was taken for comparison (Table 1). The 2θ location of diffraction peak detected in rapid laser-melted pure Mg was the same as that of the standard α-phase Mg. For Mg-1Mn, the 2θ location of the diffraction peak of Mg shifted to a higher 2θ location. Meanwhile, more significant shifts of 2θ locations were detected in the Mg-2Mn and Mg-3Mn alloys. According to Bragg’s law [23]:

2*d*·sinθ = *n*λ (*n*=1,2,3···) (1)

The shift of 2θ indicated a change of *d*, namely, the lattice plane distance, which was believed to be induced by the solid solution of Mn. When Mn dissolved in the Mg matrix, Mn atoms replaced the Mg atoms in the Mg lattice, which had an arrangement of a hexagonal close-packed structure. However, the atomic radius of Mn is larger than that of Mg; thus, the replacement of Mn atoms may lead to a crowding on the spatial location of the surrounding Mg atoms. As a consequence, the lattice spacing decreased gradually as Mn dissolved (Table 1). Moreover, the diffraction peaks of Mg in the Mg–3Mn alloy became considerably broad (Figure 2b), and the intensity decreased significantly (Table 1), which implied the formation of finer grains.

**Figure 3:**
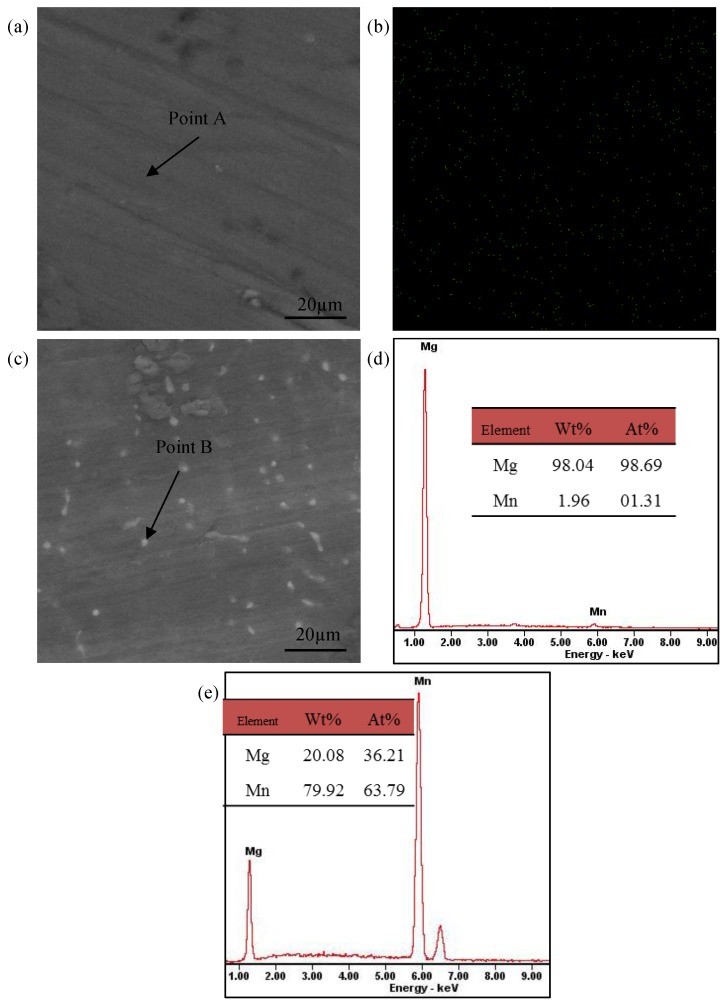
(**a**) BSE image of Mg-2Mn alloy; (**b**) EDS map of Mn element in Mg-2Mn alloy; (**c**) BSE image of Mg-3Mn alloy; (**d**) Corresponding point EDS analysis marked by arrow A in Figure 3a; (**e**) Corresponding point EDS analysis of bright grain marked by arrow B in Figure 3c.

Back scattered electron (BSE) imaging combined with energy dispersive spectroscopy (EDS) were used to further illustrate the compositional distribution of Mg-*x*Mn alloys (Figure 3). The BSE image (Figure 3a) with uniform gray level revealed that there was only one phase in the Mg-2Mn alloy. Meanwhile, the corresponding EDS point results (Figure 3d) revealed that the ratio of Mn in the Mg-2Mn matrix was 1.96 wt %, which was very close to that in the original powder (2 wt %). Additionally, the corresponding EDS map (Figure 3b) suggested that Mn was homogeneously distributed in the Mg-2Mn alloy. Additionally, there were a few bright grains, as shown in Figure 3c, unorderly distributed in the Mg-3Mn alloy. The EDS point analysis indicated that the bright grains were undissolved Mn particles (Figure 3e). The appearance of distinct gray level (intensity of BSE signal) between the Mg matrix (dark) and the Mn grains (bright) was due to the fact that Mn has a higher atomic number.

### 2.2. Mechanical Properties

**Figure 4:**
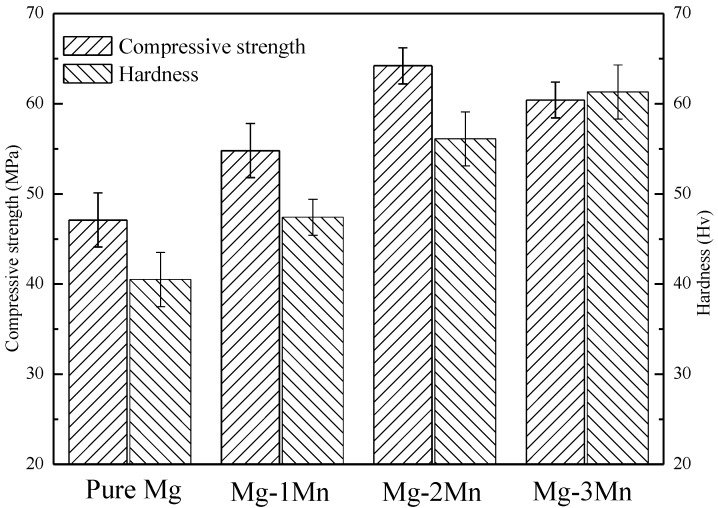
The compressive strength and hardness of Mg-Mn alloys.

The mechanical properties of rapid laser-melted Mg-*x*Mn alloys were tested (Figure 4). It can be seen that the compressive strength firstly increased from 47.1 MPa for pure Mg to 64.2 MPa for the Mg-2Mn alloy, and was followed by a decrease to 60.4 MPa with increasing Mn up to 3 wt %. The improved compression strength was partly attributed to the fine grain strengthening effect. The plastic deformation could be dispersed into more grains with the size of grains decreasing, leading to an improved ability to withstand the external force. On the other hand, the solid solution of Mn caused a crystal lattice distortion in the Mg matrix, which was expected to retard dislocation sliding during the deformation and further improved the compression strength. However, the undissolved Mn phase resulted in a weak interface between adjacent Mg and Mn grains where the cracks were easy to expand, which impaired the compressive strength of the Mg-3Mn alloy. 

In addition, as Mn increased from 0 to 3 wt %, the hardness increased from 40.5 Hv for pure Mg to 56.1 Hv for the Mg-2Mn alloy and 61.3 Hv for the Mg-3Mn alloy. The improved hardness of the alloys was due to the solid solution strengthening and fine grain strengthening. With respect to the role of grain refinement, Ng [24] revealed that laser-melted pure Mg (grain sizes of ~4 µm) obtained by specific process parameters exhibited a hardness of ~63 Hv, which was higher than that of the Mg-2Mn alloy. It was believed that grain refinement had a more dominant effect on improving the mechanical properties of Mg alloys. Moreover, the undissolved Mn phase in the Mg-3Mn alloy was also responsible for the improved hardness due to the higher hardness of Mn than that of Mg.

### 2.3. Corrosion Resistance

**Figure 5:**
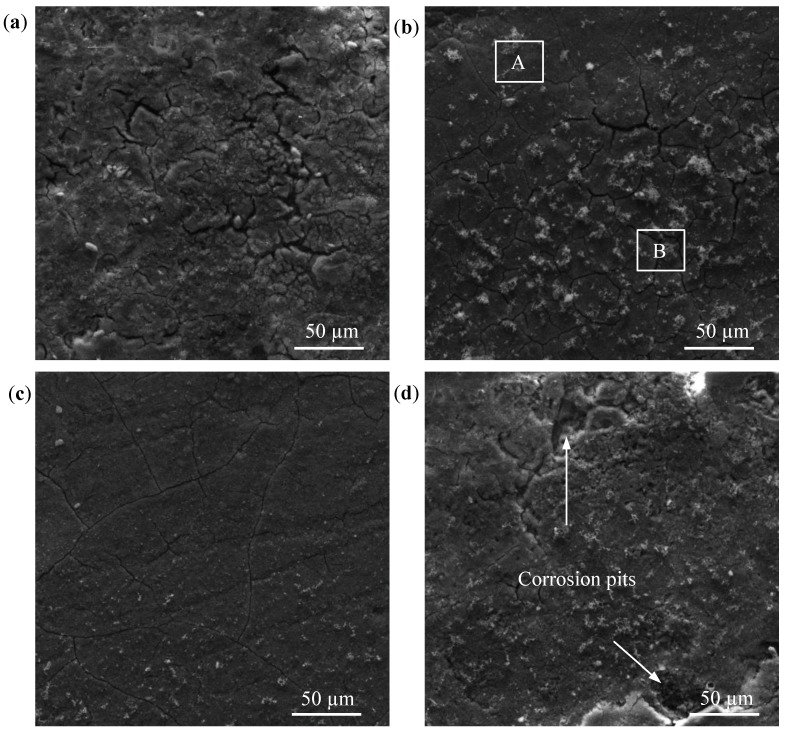
SEM micrographs of rapid laser-melted Mg-*x*Mn alloys after immersion in SBF at 37 °C for 48 h: (**a**) pure Mg; (**b**) Mg-1Mn, area A was covered with integrated protective film, while area B presented relatively shallow cracks; (**c**) Mg-2Mn; (**d**) Mg-3Mn.

The surface morphologies of the Mg-*x*Mn alloy after soaking in SBF for 48 h are shown in Figure 5. The pure Mg exhibited a cracked surface, which was covered with a large amount of corrosion product (Figure 5a). The appearance of cracks is believed to be caused by the dehydration of the corrosion product layer after drying in warm air and under the vacuum of the SEM chamber [25]. For the Mg-1Mn alloy, local areas presented relatively shallow cracks (area B in Figure 5b), while some positions (typically area A in Figure 5b) were still covered with integrated protective film. In contrast, the surface of the Mg-2Mn alloy maintained the integrity of the corrosion film, and the scratches caused by the pretreatment of the sample could be easily observed (Figure 5c), which implied that the corrosion on the surface of the Mg-2Mn alloy was relatively slight. As for the Mg-3Mn alloy, some positions on the surface were severely destroyed, and deep corrosion pits, as shown in Figure 5d, were left on the surface.

**Figure 6:**
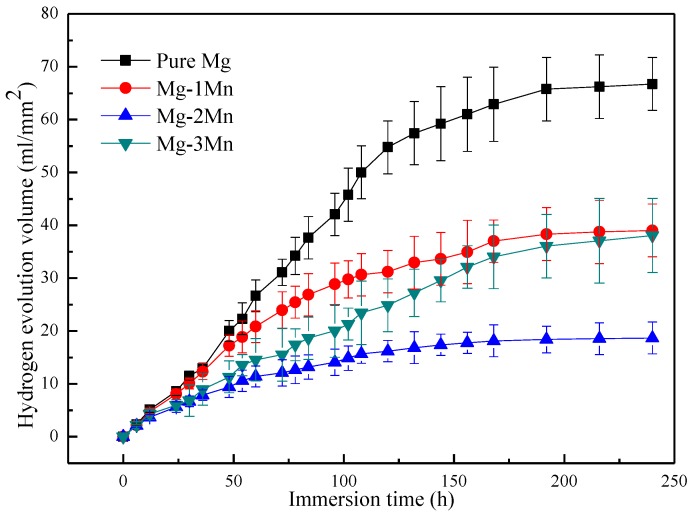
The hydrogen evolution volume of rapid laser-melted Mg-*x*Mn alloys as function of immersion time.

The hydrogen volume evolution during the soak in SBF was observed, and the results are shown in Figure 6. It indicated that the average hydrogen volume evolution rate from the Mg-2Mn alloy sample (0.017 mL·cm^−2^·h^−^^1^) was significant lower than that from the pure Mg (0.068 mL·cm^−2^·h^−^^1^), Mg-1Mn (0.041 mL·cm^−2^·h^−^^1^) and Mg-3Mn alloy samples (0.039 mL·cm^−2^·h^−^^1^). The enhanced corrosion resistance can partly be ascribed to the increase in corrosion potential caused by the solid solution of Mn, which possessed a higher corrosion potential (−1.18 V) than that of Mg (−2.34 V) [16]. As Mg alloys soak in SBF, the electrochemical reactions occurred as follows:

Anodic: Mg → Mg^2+^ + 2e (2)

Cathodic: 2H^+^ + 2e → H_2_↑ (3)

The reactions were driven by the relative potential difference between the relative anode and cathode. Thus, the increase in corrosion potential of the Mg matrix, namely, the decrease in relative potential difference, had a beneficial effect on reducing the relative anodic and cathodic reaction kinetics supported by the Mg alloy.

On the other hand, the enhancement of corrosion resistance is linked to the grain refinement. Orlov *et al.*’s study [15] on the effect of microstructure on the corrosion behavior of Mg alloys revealed that the corrosion resistance of Mg alloys is closely related to total grain boundary length, and the electrode reaction kinetics supported by the alloy deceased as the total length of the grain boundaries increases. As for the Mg-3Mn alloy, the heterogeneously distributed Mn phase served as a cathode and formed a galvanic couple with the Mg matrix, which accelerated the Mg corrosion rate. As a consequence, the susceptible Mg matrix adjacent to the Mn phase was preferentially corroded and formed some deep corrosion pits (Figure 5d).

## 3. Materials and Methods 

### 3.1. Materials

**Figure 7:**
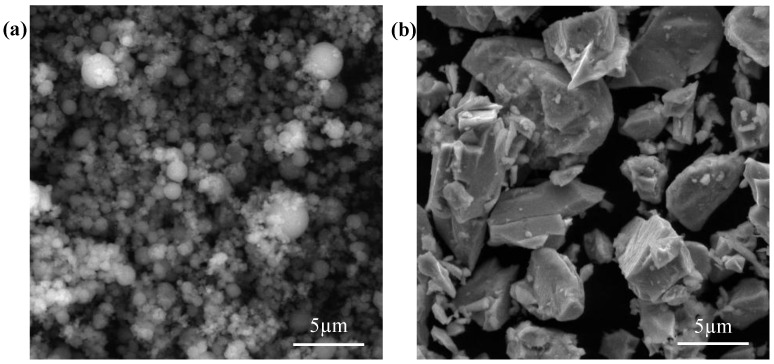
(**a**) Mg powder and (**b**) Mn powder.

The pure Mg powder (99.9%) with spherical shape (Figure 7a) and the Mn powder (99.9%) with irregular shape (Figure 7b) were purchased from Shanghai Naiou Nano technology Co., Shanghai, China. Mn powder (1, 2, and 3 wt %) were added respectively to the Mg powder, followed by mechanical mixing under a mixed gas atmosphere of SF_6_ and CO_2_. Pure Mg powder was also prepared using as reference sample.

### 3.2. Laser-Melting Process

**Figure 8:**
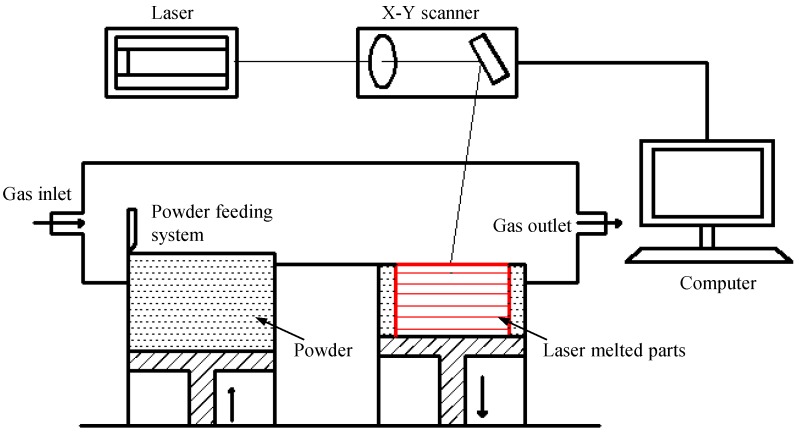
Schematic of laser-melting system.

The home-made rapid laser-melting system, as depicted in Figure 8, was composed mainly of a fiber laser with a rated power of 100 W, a powder feeding system, an argon gas protecting system, and a computer control system for process controlling. More detailed information regarding the laser-melting system can be found in [26]. Before the experiments, argon gas was fed into the sealed building chamber to provide an ideal experimental condition. A thin powder layer with a thickness of 100 µm was then paved onto the substrate (commercial Mg alloy AZ61) by the powder feeding system. Afterwards, the high energy laser beam scanned the powder layer to form a layer-wise profile according to the CAD data from the computer control system. A simple linear raster scan pattern was used with a scanning length of 4 mm and a line spacing of 50 µm. Then, the samples (4 mm × 4 mm × 2 mm), which had a smooth surface without dimensional distortion, were built up in a layer-by-layer method. The optimized process parameters: laser output power of 70 W, a laser beam scanning speed of 700 mm/min, and a laser spot diameter of 100 µm.

### 3.3. Microstructural Characterizations

The samples were ground and polished according to standard procedures and etched with a acetic-picral solution (10 mL acetic acid + 4.2 g picric acid + 70 mL ethanol) for 10 s. Optical microscopy (PMG3, Olympus Corporation, Tokyo, Japan) observations were performed to analyze the crystalline structure. Moreover, scanning electron microscopy (JSM-5600LV, JEOL Co., Tokyo, Japan) observations in BSE mode and EDS (JSM-5910LV, JEOL Co., Tokyo, Japan) analysis were performed at accelerating voltages of 5 kV and 20 kV to identify the compositional distribution.

Phase analysis was performed by XRD (D8 Advance, Bruker AXS Inc., Karlsruhe, Germany) with the copper target, Kα X-ray (λ = 1.54056 Å) at 40 kV and 40 mA. A quick scanning rate of 4°·min^−1^ was performed over a wide range of 2θ = 30°–80° to give an overview of the diffraction peaks. A slower scanning rate of 1°·min^−1^ was then performed over 2θ = 35.5°–37.5° to obtain a detailed characterization of the diffraction peaks.

### 3.4. Mechanical Tests

The compressive strength of the samples was tested by using a universal testing machine (WD-01, Shanghai Zhuoji instruments Co. Ltd., Shanghai, China) with the maximum load of 100 N and rate of 0.5 mm·min^−1^. Five measurements were carried out for per group. 

The hardness of the samples was tested on the polished surface using a HXD-1000TM/LCD Digital Micro-hardness Tester (Shanghai Taiming Optical Instrument Co., Shanghai, China) under a load of 2.942 N for 10 s. Five different locations were tested for each sample.

### 3.5. Immersion tests

The immersion tests were performed in SBF according to standard procedures [27]. The temperature was kept at 37 °C using bath water, and the pH was adjusted to 7.4. After soaking for 48 h, the samples were removed from SBF, then gently washed with ethyl alcohol, and dried at room temperature. The surface morphologies after soaking were characterized by SEM in secondary electron mode. The hydrogen evolution volume of rapid laser-melted Mg-*x*Mn alloys was monitored during the immersion. Five measurements were carried out for the average.

## 4. Conclusions

The microstructure, mechanical properties, and corrosion resistance of rapid laser-melted Mg-*x*Mn (*x* = 0–3 wt %) alloys were investigated. An addition of 2 wt % Mn dissolved completely in the Mg matrix inducing the crystal lattice distortion during the rapid solidification process. Additionally, both mechanical properties and corrosion resistance were enhanced. The Mg-2Mn alloy had an improved compressive strength of 64.2 MPa and a hardness of 56.1 Hv, respectively. The enhancement of Mg corrosion resistance could be attributed to the increase in corrosion potential and grains refinement caused by the solid solution of Mn. It is suggested that rapid laser-melted Mg-2Mn alloy is a potential candidate for future bone implants.

## Figures and Tables

**Table 1:** The location, lattice spacing, and intensity variation of identified diffraction peak of Mg phase.

Sample	Standard	Pure Mg	Mg-1Mn	Mg-2Mn	Mg-3Mn
2θ location (°)	36.53	36.53	36.59	36.71	36.72
Lattice spacing (Å)	2.45772	2.45772	2.45382	2.44608	2.44543
Intensity (CPS)	-	5709	5309	5016	4220
